# Supplementary material for: Large Stokes shift fluorescence activation in an RNA aptamer by intermolecular proton transfer to guanine
Source: Nat Commun. 2021 Jun 10;12:3549. doi: 10.1038/s41467-021-23932-0 (PMC8192780; doi:10.1038/s41467-021-23932-0)
Supplement: Supplementary file 1 — Supplementary Information [file 41467_2021_23932_MOESM1_ESM.pdf]

Supplementary Information

Large Stokes shift fluorescence activation in an RNA aptamer by intermolecular proton transfer to guanine

Mateusz Mieczkowski,[#] Christian Steinmetzger,[#] Irene Bessi, Ann-Kathrin Lenz, Alexander Schmiedel, Marco Holzapfel, Christoph Lambert,* Vladimir Pena* and Claudia Höbartner*

# equally contributing authors. * corresponding authors

Table of Contents

Supplementary Tables 1-3

Supplementary Figures 1-20

Supplementary References

Supplementary Table 1 | Crystallographic data table.

	Chili–DMHBI$^+$ complex (Crystal I)	Chili–DMHBO$^+$ complex (Crystal II)	Chili–DMHBO$^+$ Iridium co-crystallized form (Crystal III)	Chili–DMHBO$^+$ Iridium soaked form (Crystal IV)
Pdb code	7OAW	7OAX	7OA3	7OAV
Data collection				
Wavelength (Å)	1.0332	0.9998	1.1035	1.1048
Resolution range [a]	49.48 - 2.89 (3.06 - 2.89)*	40.97 - 2.24 (2.34 - 2.24)	45.92 - 2.80 (2.95 - 2.80)	46.14 - 2.85 (3.00 - 2.85)
No. mol/AU	4	4	2	4
Space group	P 2_1 2_1 2_1	P 2_1 2_1 2_1	I 1 2 1	P 1 2_1 1
Unit Cell				
a, b, c (Å)	59.1 100.0 113.9	59.3 100.4 113.3	70.6 54.1 87.5	57.3 106.8 63.6
α, β, γ (˚)	90 90 90	90 90 90	90 95.5 90	90 116.5 90
R-merge	0.2 (>1.0)	0.04 (0.95)	0.07 (>1.0)	0.12 (>1.0)
Unique reflections	15716 (2474)	25428 (1271)	8215(1177)	15836 (2270)
Mean I/σI	8.2 (0.4)	19.3 (1.9)	13.0 (0.6)	8.6 (0.4)
Redundancy	13.0 (12.6)	6.6 (6.3)	13.4 (11.7)	7.1 (7.3)
Completeness (%) (Spherical)	99.7 (99.0)	76.7 (24.9)	99.7 (98.0)	98.7 (97.7)
Completeness (%) (Ellipsoidal)	-	94.5 (91.5)	-	-
CC1/2 (%)	99.9 (31.7)	100 (80.6)	100 (53.4)	99.8 (21.2)
Refinement				
Resolution range	46.45 - 2.95	40.98 - 2.25	45.92 - 2.80	46.14 - 2.99
R-work/R-free (%)	21.8/26.5	20.2/23.7	22.5/24.1	21.8/25.6
No. reflections	14204 (986)	25382 (706)	8166 (780)	15583 (1400)
No. of non-H atoms	4651	4773	2307	4655
Macromolecules	4378	4400	2170	4389
Ligands/Ions	273	336	137	264
Water	0	37	0	2
Average B-factor	103.6	68.5	155.5	151.2
RNA	102.5	68.4	153.6	149.6
Fluorophore	93.8	52.5	162.5	142.1
Ligands/ions	111.2	69.6	197.6	151.5
Water	-	53.8	-	85.2
r.m.s deviations				
Bond lengths (Å)	0.001	0.002	0.013	0.002
Bond angles (°)	0.46	0.54	0.63	0.65
Coord. Precision [b] (Å)	0.56	0.34	0.60	0.55

[a] Statistics for the highest-resolution shell are shown in parentheses.
[b] Calculated by Maximum Likelihood Estimation

Supplementary Table 2 | RNA Sequences

	description	5′-sequence-3′ (RNA prepared by *in vitro* transcription)
Tr1	Parent Chili	GGCUAGCUGGAGGGGCGCCAGUUCGCUGGUGGUUGGGUGCGGUCGGCUAGCC
Tr2	Chili P1C	GGCUAGCCGGAGGGGCGCCAGUUCGCUGGUGGUUGGGUGCGGUCGGCUAGCC
Tr3	Chili P1A	GGCUAGCUGGAGGGGCGCCAGUUCGCUGGUGGUUGGGUGCGGUCAGCUAGCC
Tr4	Chili P1-1	GGCAGCUGGAGGGGCGCCAGUUCGCUGGUGGUUGGGUGCGGUCGGCUGCC
Tr5	Chili P1-2	GGAGCUGGAGGGGCGCCAGUUCGCUGGUGGUUGGGUGCGGUCGGCUCC
Tr6	Chili P1-3	GGACUGGAGGGGCGCCAGUUCGCUGGUGGUUGGGUGCGGUCGGUCC
Tr7	Chili P1A-1	GGCAGCUGGAGGGGCGCCAGUUCGCUGGUGGUUGGGUGCGGUCAGCUGCC
Tr8	Chili P1A-2	GGAGCUGGAGGGGCGCCAGUUCGCUGGUGGUUGGGUGCGGUCAGCUCC
Tr9	Chili P1A-3	GGACUGGAGGGGCGCCAGUUCGCUGGUGGUUGGGUGCGGUCAGUCC
Tr10	Chili P1A-1 circ. perm.	GGGUGCGGUCAGUCCGGAAGGACUGGAGGGGCGCCAGUUCGCUGGUGGUU
Tr11	µChili	GGACUGGAGGGGCGCCGGAAGGUGGUUGGGUGCGGUCGGUCC
Tr12	Chili G15A	GGCUAGCUGGAGGGACGCCAGUUCGCUGGUGGUUGGGUGCGGUCGGCUAGCC
Tr13	Chili C40U	GGCUAGCUGGAGGGGCGCCAGUUCGCUGGUGGUUGGGUGUGGUCGGCUAGCC
Tr14	Chili G15A/C40U	GGCUAGCUGGAGGGACGCCAGUUCGCUGGUGGUUGGGUGUGGUCGGCUAGCC
	description	5'-sequence-3' (RNA prepared by solid-phase synthesis)
R1	Chili native 5' fragment	GGCUAGCUGGAGGGGCGCCAGUUCGC
R2	Chili native 3' fragment	pUGGUGGUUGGGUGCGGUCGGCUAGCCp
R3	Chili c^7G15 5' fragment	GGCUAGCUGGAGGGXCGCCAGUUCGC | X = c^7G
R4	Chili U34X 3' fragment	pUGGUGGUXGGGUGCGGUCGGCUAGCCp | X = r4Cl
R5	Chili G46X 3' fragment	pUGGUGGUUGGGUGCGGUCGXCUAGCCp | X = r4Cl

Supplementary Table 3. **|** Comparison of chromophore torsion angles[a] in fluorogenic aptamers and exemplary LSS FPs.

	pdb	τ / °	φ / °
DFHBI in Spinach	4TS0	-3.5	3
DFHO in Corn	5BJO	-2	-3
DMHBI$^+$ in Chili	7OAW	18	-41
DMHBO$^+$ in Chili	7OAX	11	-27
LSSmKate1	3NT9	6	-20
mCRISPred	6XWY	27	-30

[a] see Supplementary Figure 4 for definition of torsion angles τ and φ.

Supplementary Figures

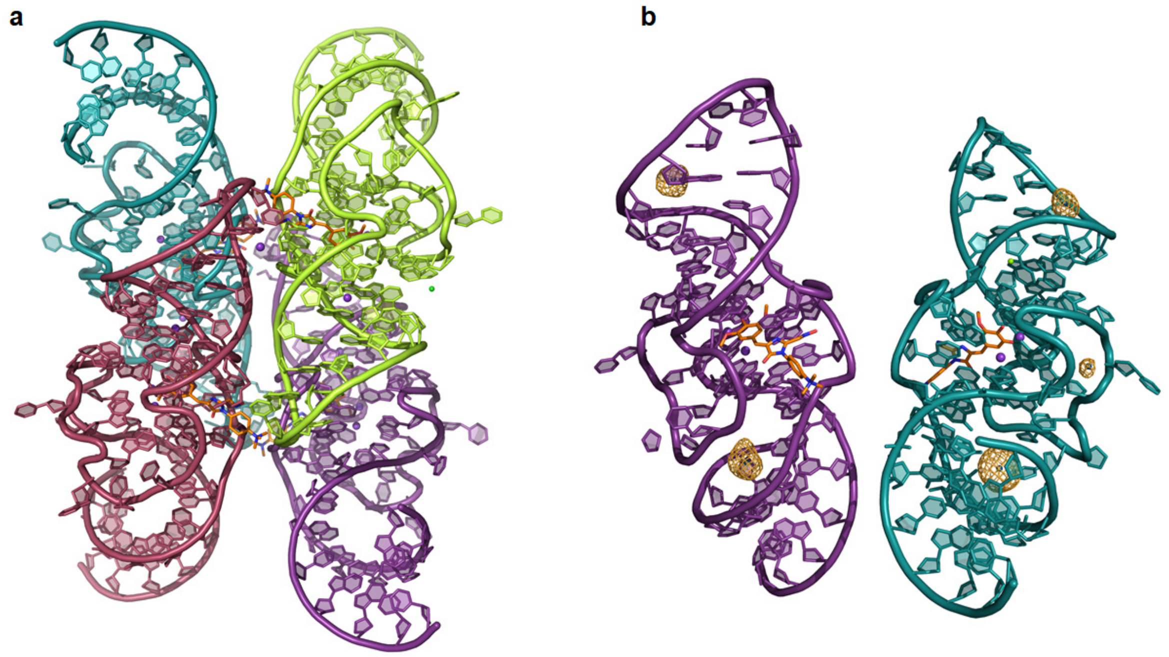

Supplementary Figure 1 | Overall crystal structure of Chili RNA aptamer

(a) Overall crystal structure of Chili RNA aptamer in complex with $DMHBO^{+}$. The ASU contains 4 copies of the RNA-ligand complex. (b) Overall crystal structure of Chili RNA aptamer in complex with $DMHBO^{+}$ co-crystallized with Iridium (III) hexamine (2 copies in the unit cell). Yellow mesh indicates anomalous difference Fourier map contoured at 5σ. The difference map was computed from data collected at the Ir L-III edge.

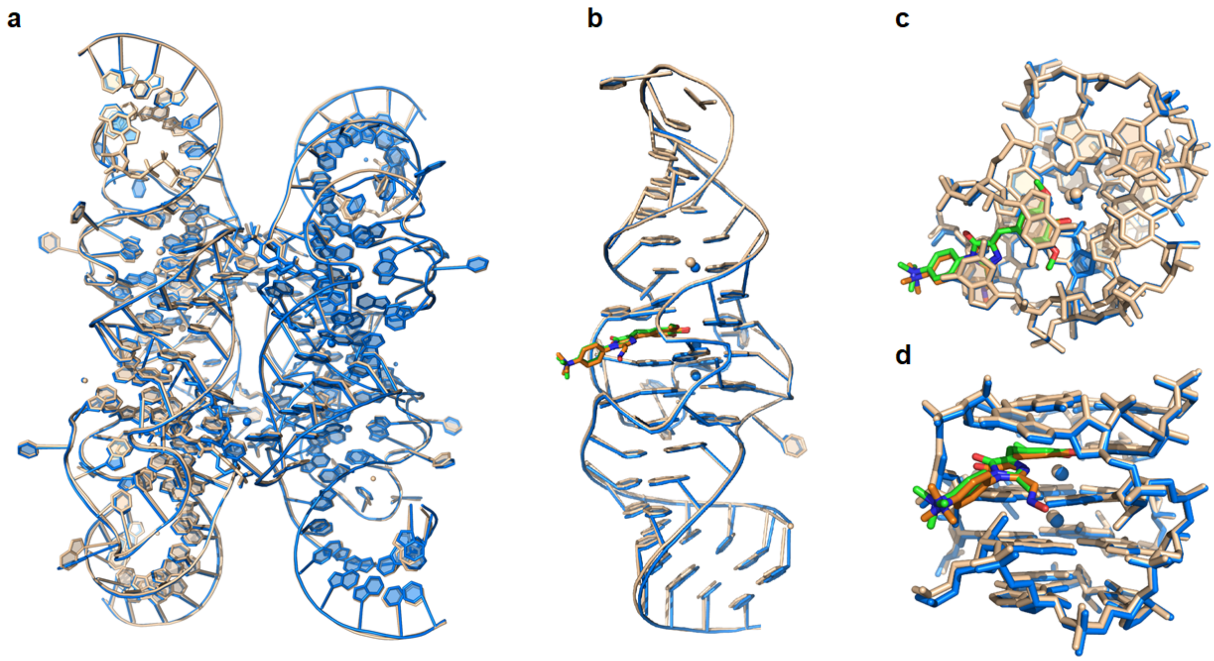

Supplementary Figure 2 | Comparison of Chili−$DMHBO^{+}$ and Chili−$DMHBI^{+}$ complexes

(a) Superimposed molecules in the asymmetric unit of the Chili RNA aptamer. (b) Superimposed single chains of Chili RNA aptamer with bound fluorophores. (c, d) Top and side view for the fluorophore binding site. Chili RNA is represented in blue for $DMHBO^{+}$ complex and in beige for $DMHBI^{+}$ complex. $DMHBO^{+}$ and $DMHBI^{+}$ fluorophores are shown in stick representation, coloured in orange and green respectively.

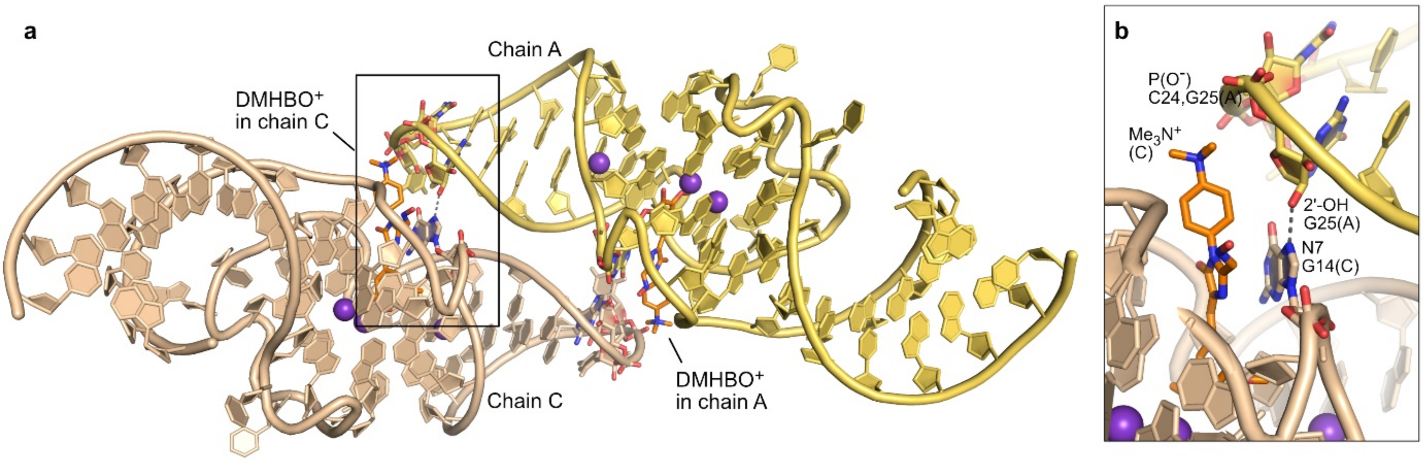

Supplementary Figure 3 | Intermolecular contacts of Chili RNA ligand complexes
(a) Electrostatic interaction of positively charged ligand sidechain with phosphate backbone of tetraloop in neighboring complex, and an intermolecular H-bond between the 2'-OH of G25 with N7 of G14 (O-N distance 2.6 Å) in the neighboring complex. (b) close-up view of boxed region in a).

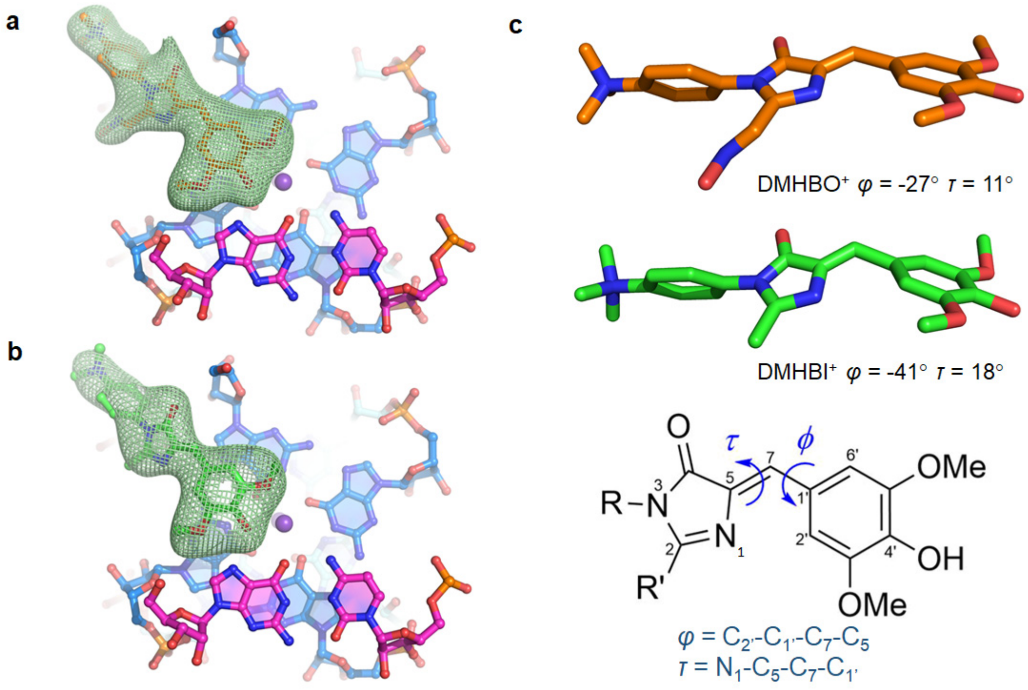

Supplementary Figure 4 | Comparison of DMHBO$^+$ and DMHBI$^+$ binding sites
Top view of (a) DMHBO$^+$and (b) DMHBI$^+$ binding site. The green mesh indicates polder omit map contoured at 4σ. (c) Definition of torsion angles τ and φ, and representative DMHBO$^+$ / DMHBI$^+$ ligand conformations in chain A.

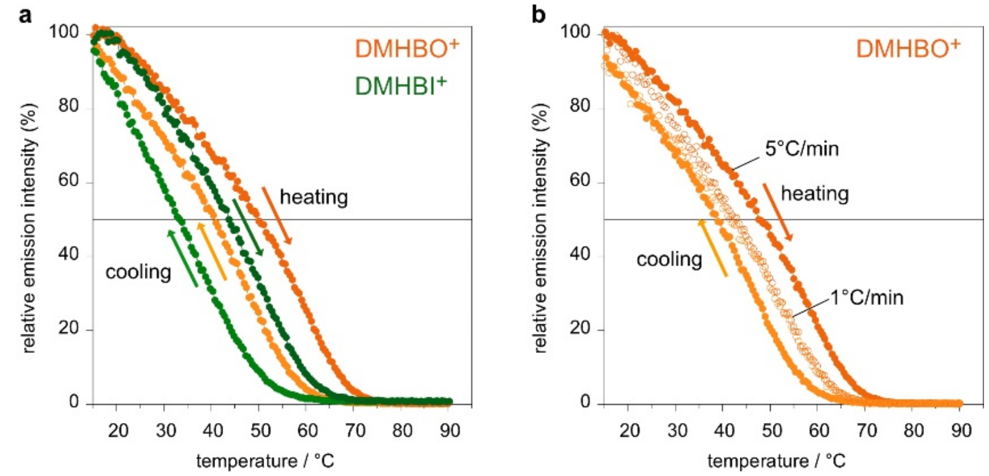

Supplementary Figure 5 | Fluorescence melting curves of Chili–DMHBO$^+$ and Chili–DMHBI$^+$. a) heating and cooling ramps recorded at 5 °C/min, both show ca. 6 – 7 °C higher $T_{1/2}$ (50% loss of initial fluorescence) for the DMHBO$^+$ complex (orange) compared to the DMHBI$^+$ complex (green). DMHBI$^+$: λ_{ex} = 415 nm, λ_{em} = 530 nm; DMHBO$^+$: λ_{ex} = 455 nm, λ_{em} = 600 nm; b) The hysteresis disappeared at a slower heating/cooling rate of 1 °C/min.

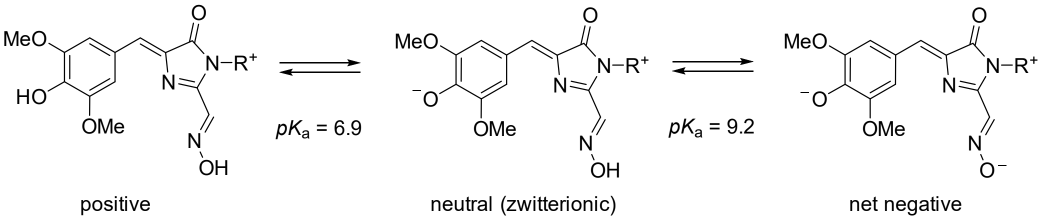

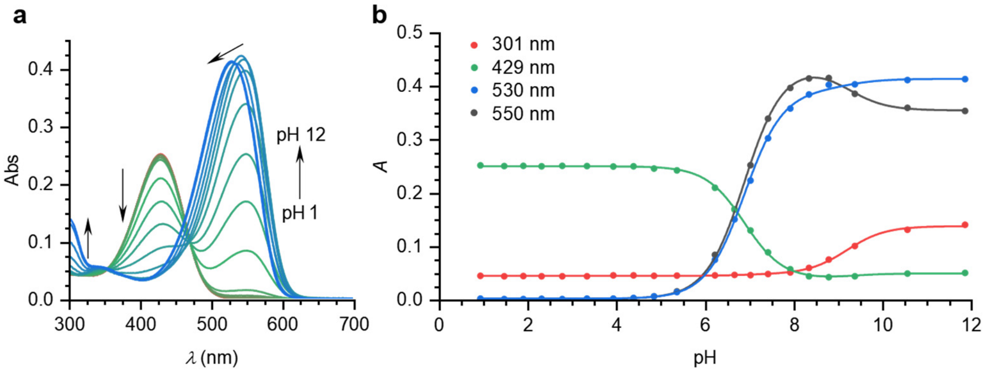

Supplementary Figure 6 | Protonation-deprotonation equilibria of DMHBO$^+$. a) UV/Vis spectra and b) titration curve at four selected wavelengths in aqueous buffer. Spectra were measured at 20 data points between pH 1 and pH 12. pK_a values were obtained by global fitting of the absorbance with the Henderson-Hasselbalch equation, and resulted in values of 6.9 for the phenol group and 9.2 for the oxime. Conditions: 11 µM ligand, 30 mM Britton-Robinson buffer. Absorbance at four individual wavelengths is plotted versus pH for one representative titration experiments (n = 3).

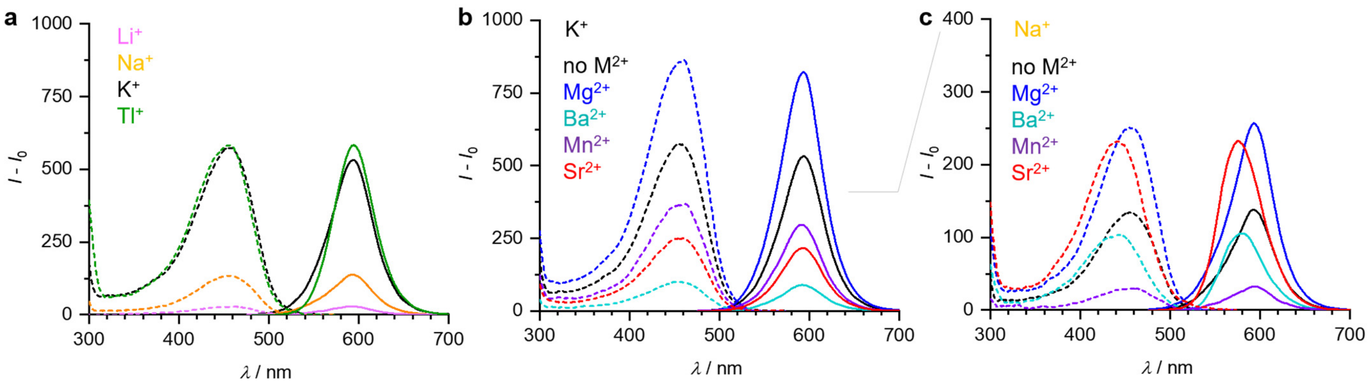

Supplementary Figure 7 | Metal ion dependence of Chili–DMHBO$^+$ fluorescence. a) Excitation and emission spectra of Chili–DMHBO$^+$ only with monovalent ions (125 mm LiCl / NaCl / KCl / TlOAc). b) Excitation and emission spectra of Chili–DMHBO$^+$ with different divalent metal ions (5 mM $MgCl_2$ / $BaCl_2$ / $MnCl_2$ / $SrCl_2$) in the presence of b) K^+ (125 mM KCl) and c) Na^+ (125 mM NaCl). Conditions: 0.5 µM RNA, 0.5 µM DMHBO$^+$, 125 mM M^+, 5 mM M^{2+}, 40 mM HEPES pH 7.5, emission spectra (solid line): λ_{ex} = 456 nm, excitation spectra (dashed line): λ_{em} = 594 nm.

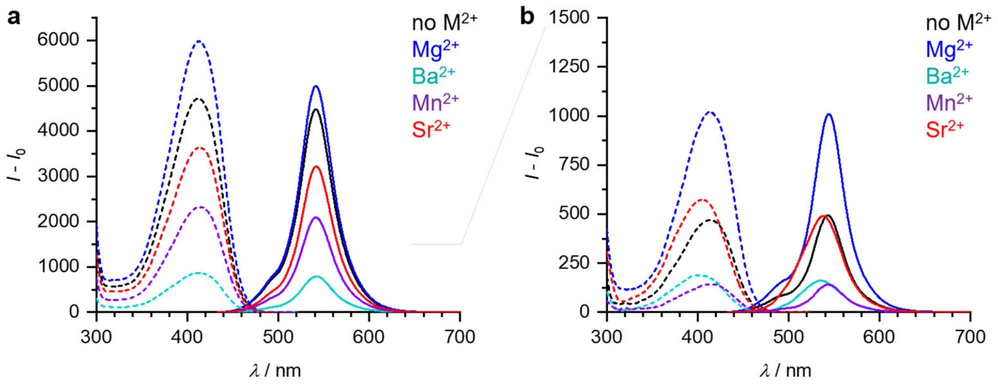

Supplementary Figure 8 | Metal ion dependence of Chili–DMHBI$^+$ fluorescence. a) Excitation and emission spectra of Chili–DMHBI$^+$ with different divalent metal ions in the presence of a) K^+ and b) Na^+. Conditions: 0.5 µM RNA, 0.5 µM DMHBO$^+$, 125 mM KCl/NaCl, 5 mM $MgCl_2$/$BaCl_2$/$MnCl_2$/$SrCl_2$, 40 mM HEPES pH 7.5, emission spectra (solid line): λ_{ex} = 413 nm, excitation spectra (dashed line): λ_{em} = 540 nm.

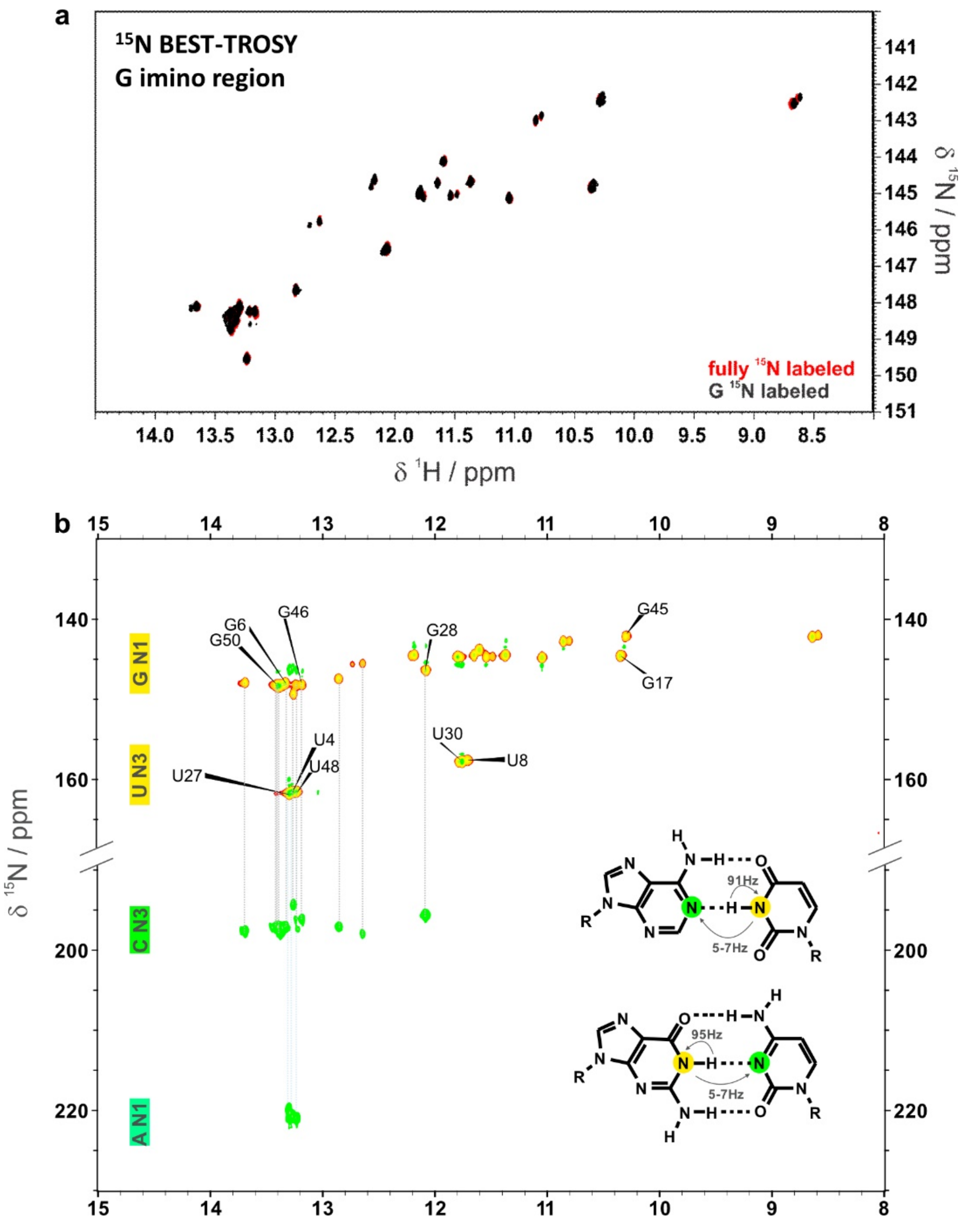

Supplementary Figure 9 | NMR spectroscopy of Chili–DMHBI$^+$ complex. a) Overlay of the guanine imino region of the ^{1}H,^{15}N-BEST-TROSY recorded on a fully ^{15}N labelled RNA in complex with the ligand (red spectrum) and on a ^{15}N guanine labelled RNA in complex with the ligand (black spectrum). b) HNN-COSY spectrum recorded on a fully ^{15}N labelled RNA in complex with the ligand with partial assignment at 700 MHz, 15 °C. By comparison of the ^{1}H,^{15}N-BEST-TROSY on the two samples the unusual signal can be assigned to a guanine. The HNN-COSY experiment rules out that the signal in question is a shifted N1 imino proton of a canonical G:C Watson-Crick base pair. Thus, the signal in question is compatible with a protonated N7 of a guanine in direct H-bonding contact with the ligand. Experimental conditions: 450 µM fully ^{15}N labelled RNA, 730 µM guanine ^{15}N labelled RNA or 380 µM unlabelled RNA in 10% D_2O/90% H_2O containing 25 mM Tris-HCl (pH 7.4), 50 mM KCl, 1 equivalent DMHBI$^+$, 25 °C, 600 MHz, unless otherwise stated.

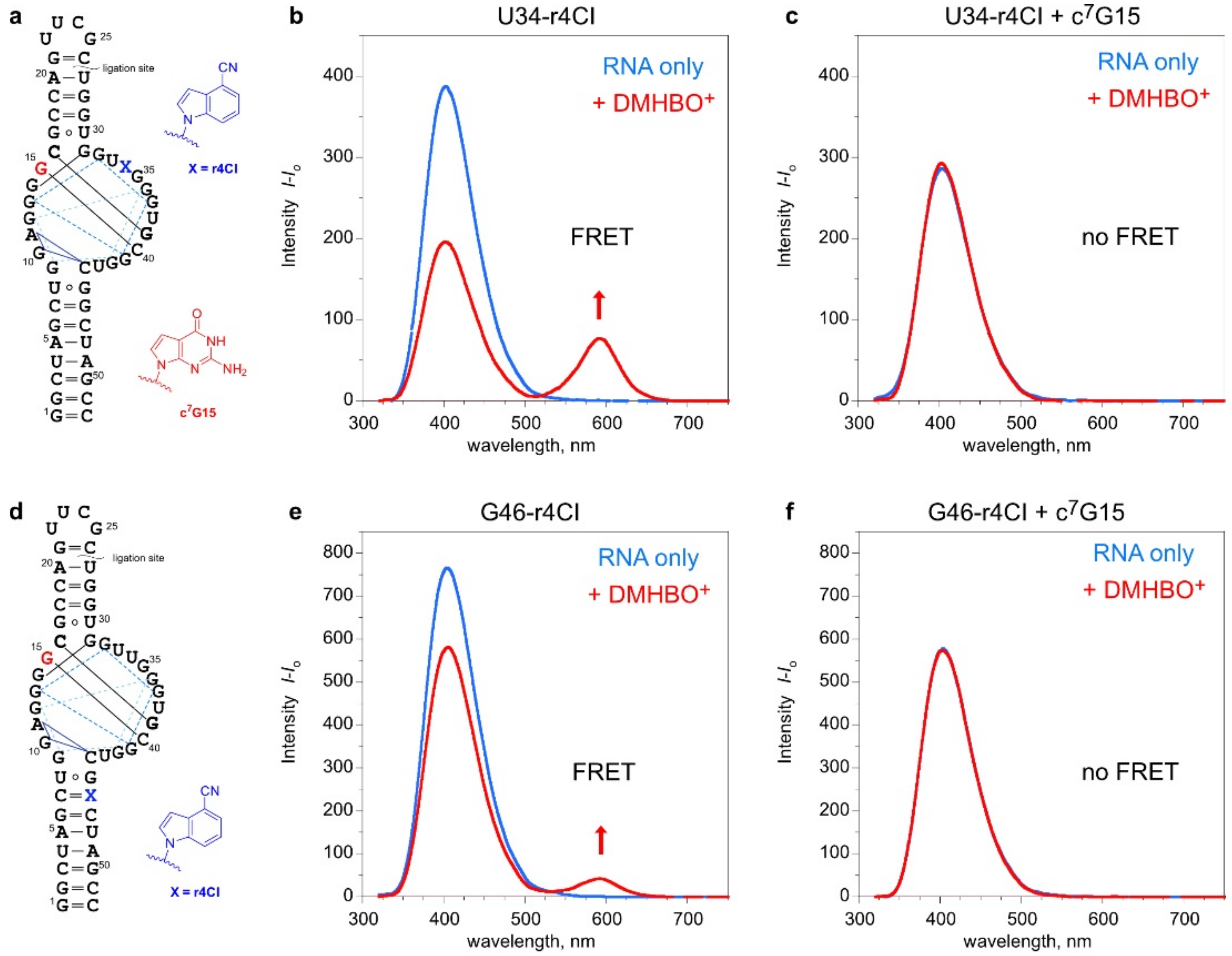

Supplementary Figure 10 | Supramolecular FRET of 4-cyanoindol-modified RNA and $DMHBO^+$ confirms that c^7G15 abolishes ligand binding. a-c) U34X, d-f) G46X; a,d) schematic secondary structure of the RNA, with modified positions indicated. b,e) reference experiments with r4CI and $DMHBO^+$ confirming binding by partial quenching of the donor fluorescence (emission at ca. 400 nm) and supramolecular FRET (emission at 592 nm). c,f) RNAs containing c^7G15 neither show any quenching of the donor nor FRET emission from the acceptor, indicating that $DMHBO^+$ cannot bind c^7G15 RNA. 0.5 µM RNA, 125 mM KCl, 5 mM $MgCl_2$, 40 mM HEPES pH 7.5, + 1 µM ligand, λ_{ex} = 303 nm.

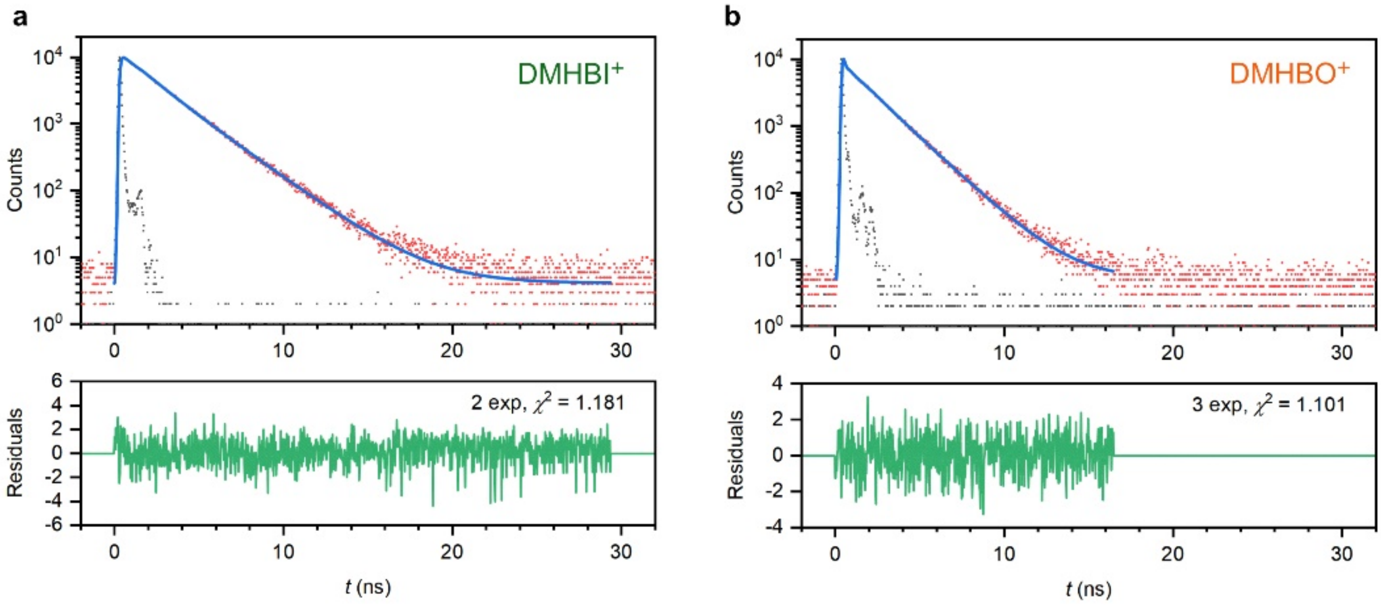

Supplementary Figure 11 | Fluorescence lifetime of (a) Chili–DMHBI$^+$ ($\lambda_{ex/em}$ = 408/542 nm) and (b) Chili–DMHBO$^+$ ($\lambda_{ex/em}$ = 477/592 nm). 0.5 µM RNA, 1 µm ligand, 125 mM KCl, 5 mM $MgCl_2$, 40 mM HEPES pH 7.5, recorded at 298 K. The decay curves (red) were fitted with two (DMHBI$^+$) or three (DMHBO$^+$) exponential decay components by iterative reconvolution with the instrument response function (black). The fit quality was assessed based on the random distribution of the residuals (green) around 0 and a χ^2 value < 1.2. The two lifetime components for Chili-DMHBI+ in a) were 2.5 ns (65%) and 1.5 ns (35%), with a ratio of amplitudes of 1.8. For Chili-DMHBO+, the two major components of the fit were 0.43 ns (80%) and 1.9 ns (14%), with a ratio of amplitudes of 5.7.

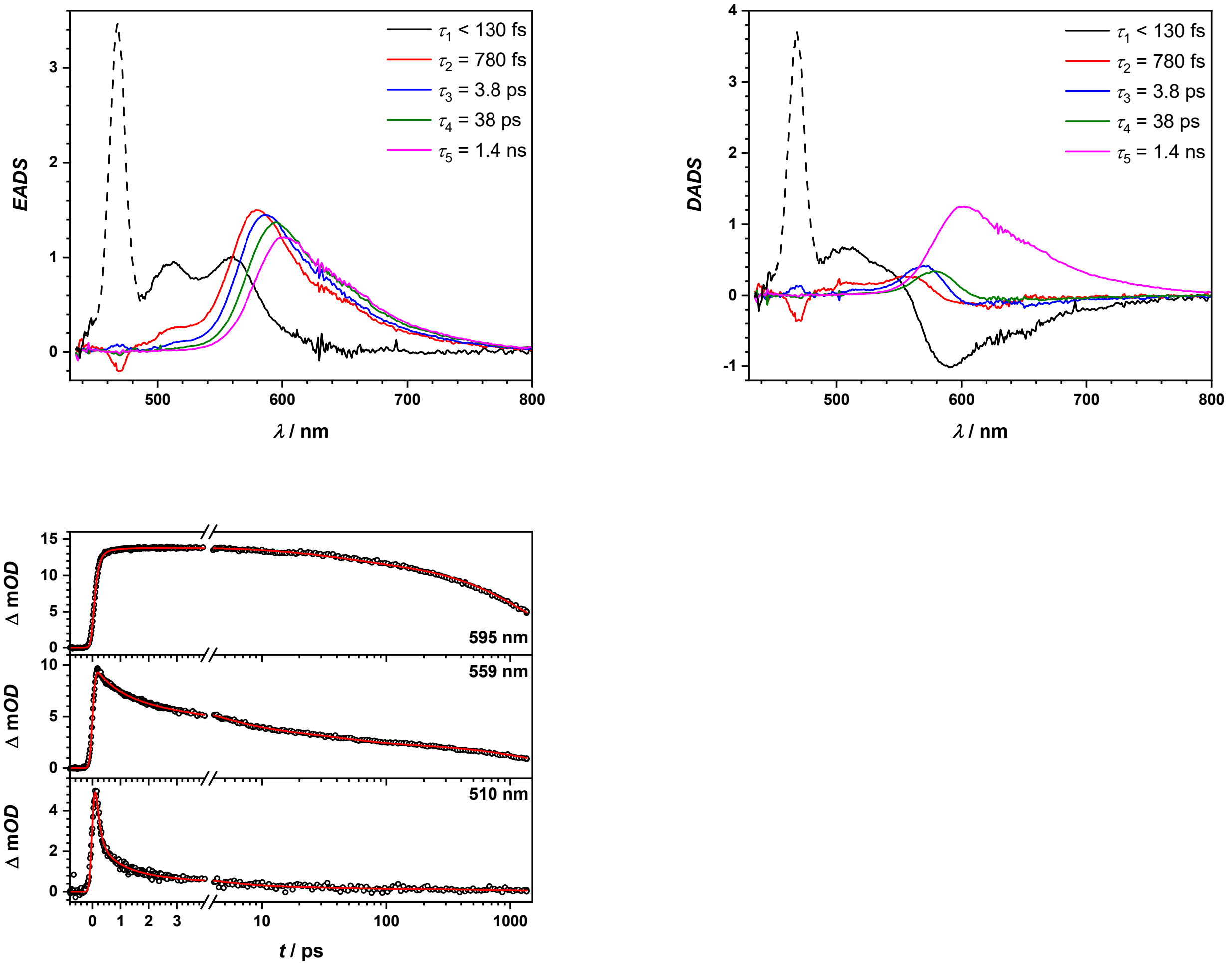

Supplementary Figure 12 | Broadband fluorescence upconversion spectra of Chili–DMHBO$^+$ after excitation at 405 nm (24700 cm^{-1}) with magic angle orientation of the pump pulse relative to the gate pulse orientation. Top left: Evolution-associated difference spectra from a global exponential fit. Top right: Decay associated difference spectra from the same global exponential fit. The sharp peak at 468 nm (21300 cm^{-1}) in the shortest component is a Raman signal of the buffer. Bottom left: Time scans and fit (red line) at selected wavelengths.

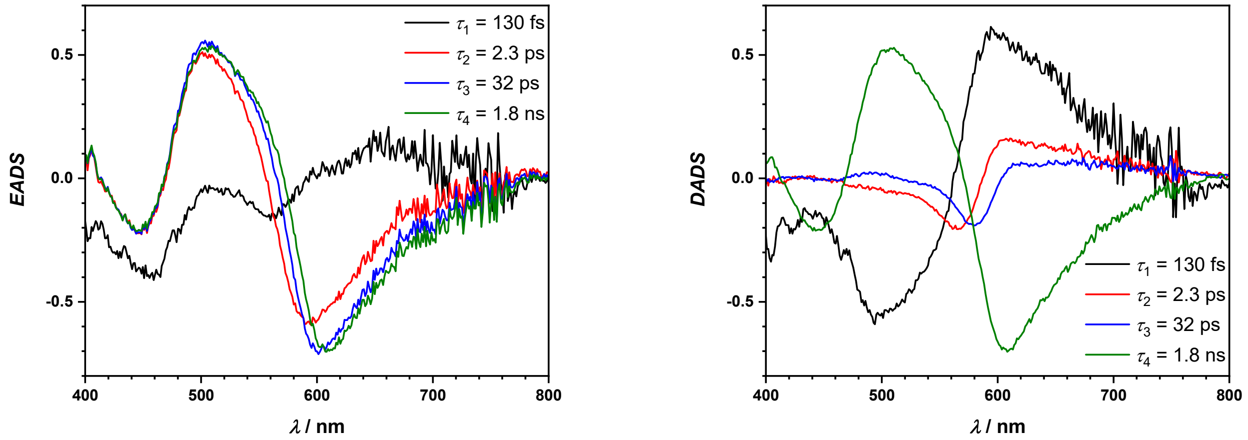

Supplementary Figure 13 | Left: Evolution associated difference spectra and Right: decay associated difference spectra from the same global exponential fit of the transient data of Chili–DMHBO$^+$ after excitation at 405 nm (24700 cm^{-1}).

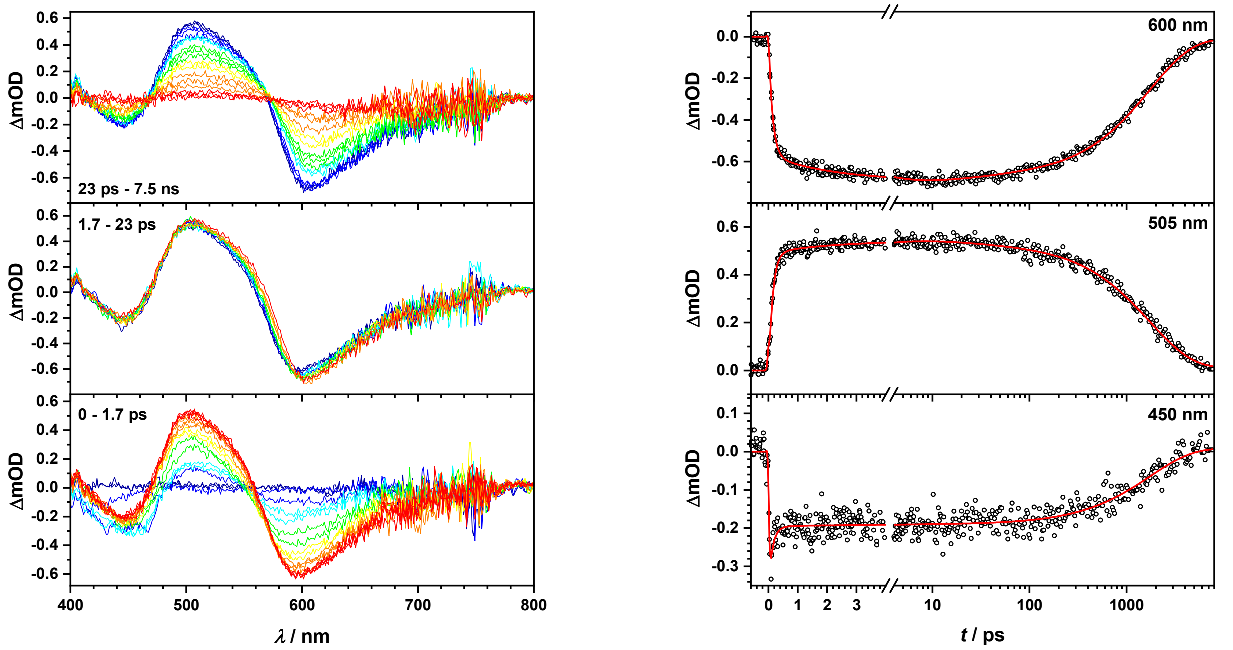

Supplementary Figure 14 | Left: Chirp and stray light corrected transient absorption spectra of Chili–DMHBO$^+$ after excitation at 405 nm (24700 cm^{-1}). In each panel, the colors change from blue to red with increasing time. Right: Time scans and fit (red line) at selected wavelengths.

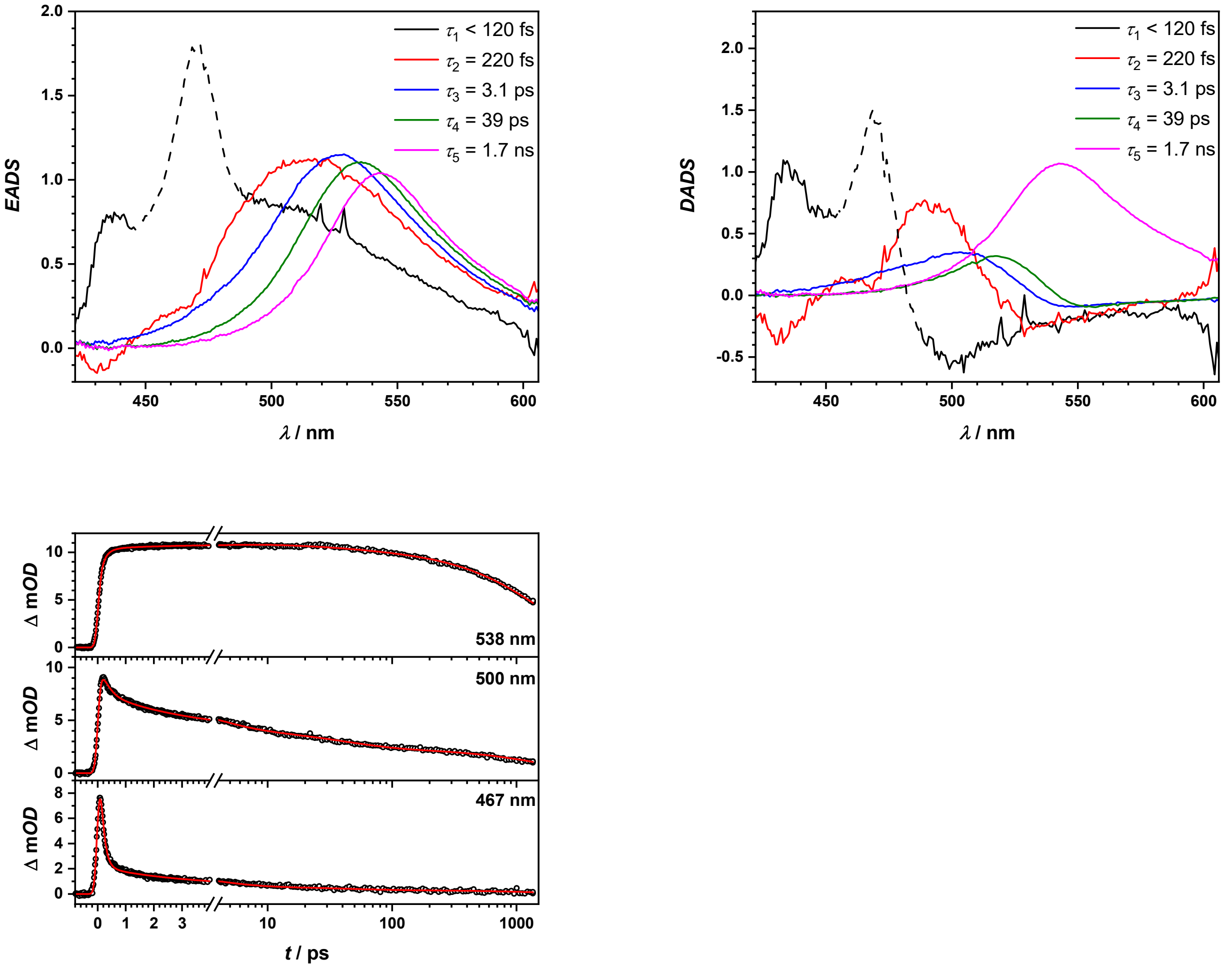

Supplementary Figure 15 | Broadband fluorescence upconversion spectra of Chili–DMHBI$^+$ after excitation at 405 nm (24700 cm^{-1}) with magic angle orientation of the pump pulse relative to the gate pulse orientation. Top left: Evolution-associated difference spectra from a global exponential fit. Top right: Decay associated difference spectra from the same global exponential fit. The sharp peak at 470 nm (21300 cm^{-1}) in the shortest component is a Raman signal of the buffer. Bottom left: Time scans and fit (red line) at selected wavelengths.

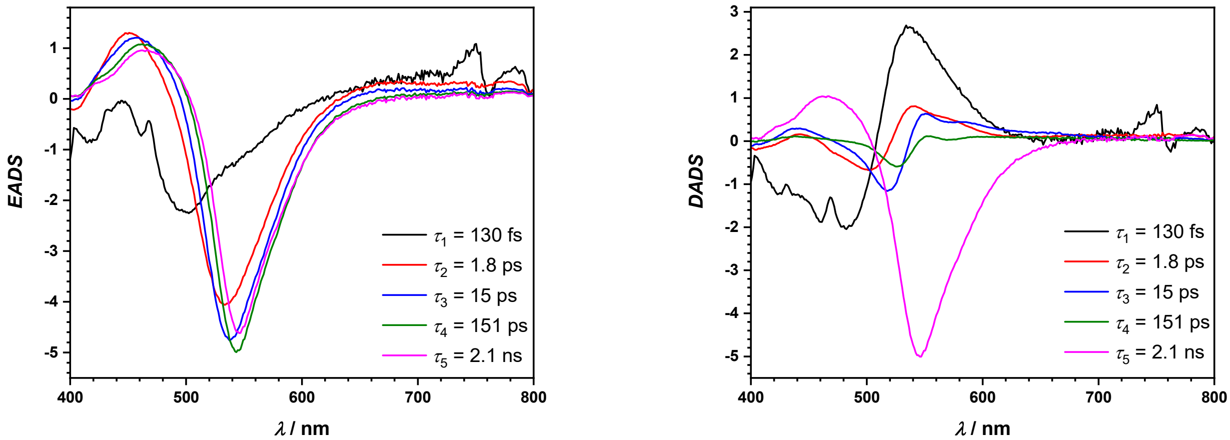

Supplementary Figure 16 | Left: Evolution associated difference spectra and Right: decay associated difference spectra from the same global exponential fit of the transient data of Chili–DMHBI$^+$ after excitation at 405 nm (24700 cm^{-1}).

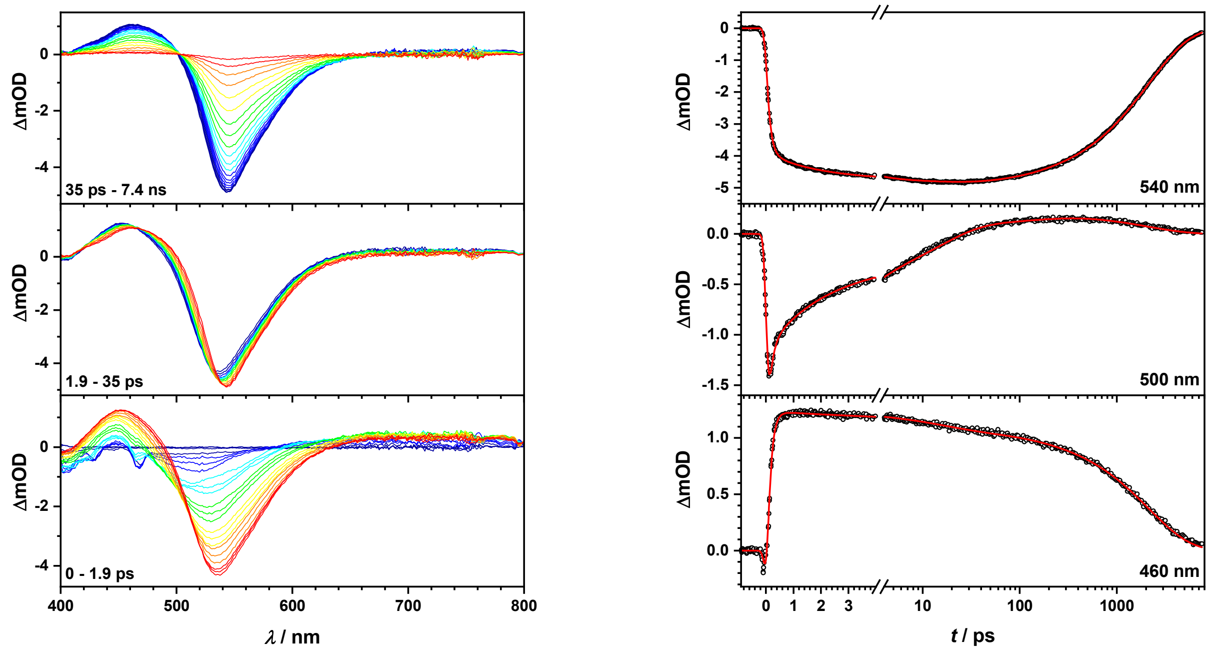

Supplementary Figure 17 | Left: Chirp and stray light corrected transient absorption spectra of Chili–DMHBI$^+$ after excitation at 405 nm (24700 cm^{-1}). In each panel, the colors change from blue to red with increasing time. Right: Time scans and fit (red line) at selected wavelengths.

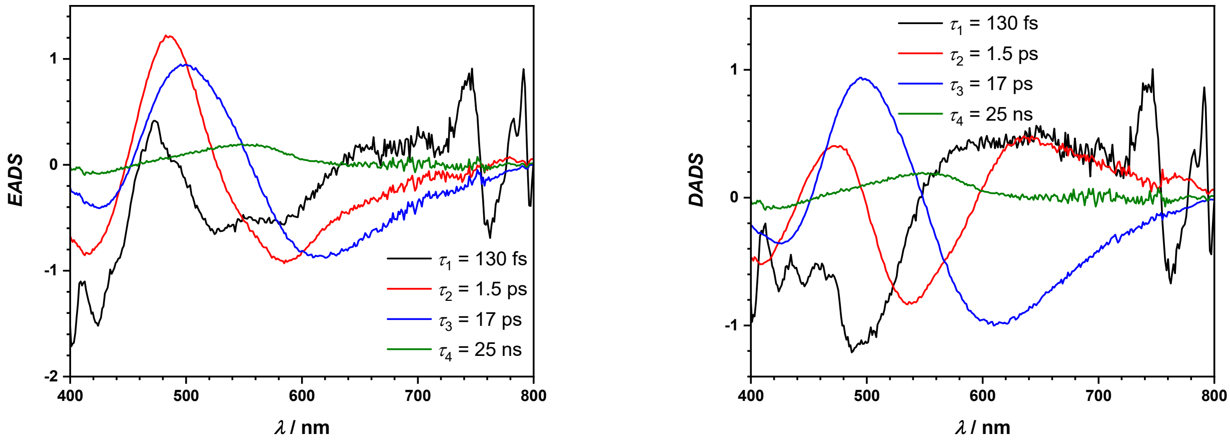

Supplementary Figure 18 | Left: Evolution associated difference spectra and Right: decay associated difference spectra from the same global exponential fit of the transient data of free DMHBO$^+$ (in 125 mM KCl, 40 mM HEPES pH 7.5, 5 mM $MgCl_2$) after excitation at 405 nm (24700 cm^{-1}). A minor but long lived (τ > 10 ns) component suggests the formation of a photoisomerized product, but its lifetime could not be measured accurately.

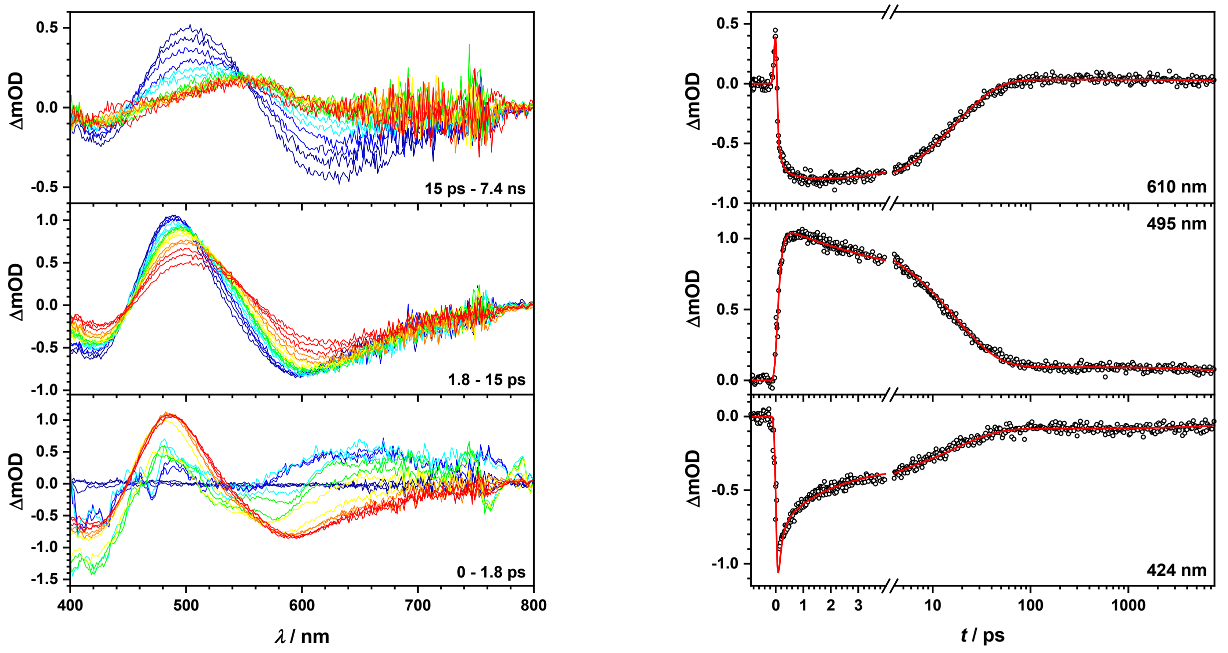

Supplementary Figure 19 | Left: Chirp and stray light corrected transient absorption spectra of free DMHBO$^+$ (in 125 mM KCl, 40 mM HEPES pH 7.5, 5 mM $MgCl_2$) after excitation at 405 nm (24700 cm^{-1}). In each panel, the colors change from blue to red with increasing time. Right: Time scans and fit (red line) at selected wavelengths.

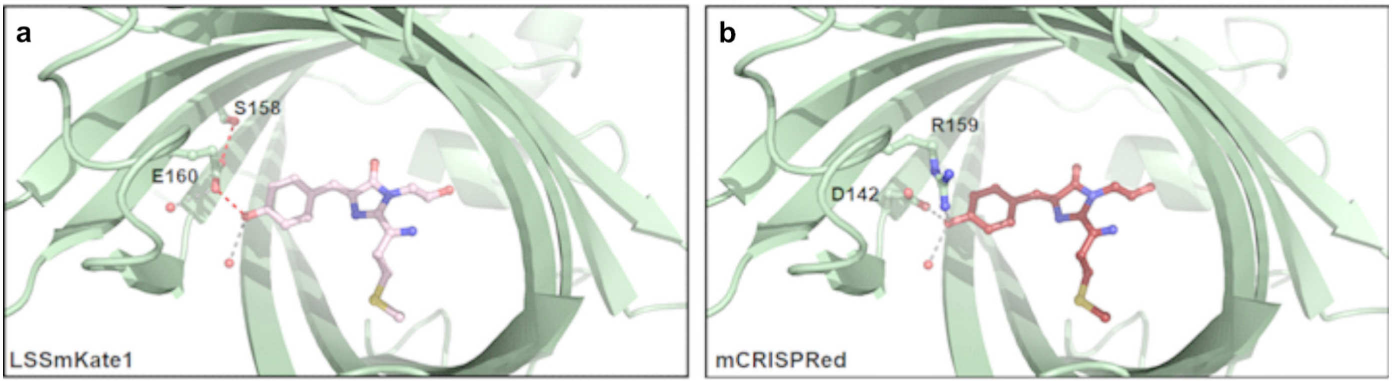

Supplementary Figure 20 | Fluorophore conformations in large Stokes shift fluorescent proteins. (a) LSSmKate1 (PDB 3NT9, chain A, $\varphi = -20°$ and $\tau = 6°$)[1] and (b) mCRISPRed (PDB 6XWY, chain A, $\varphi = -30°$ and $\tau = 27°$).[2]

Supplementary References

1 Piatkevich, K. D., Malashkevich, V. N., Almo, S. C. & Verkhusha, V. V. Engineering ESPT pathways based on structural analysis of LSSmKate red fluorescent proteins with large Stokes shift. *J Am Chem Soc* **132**, 10762-10770 (2010).

2 Erdogan, M., Fabritius, A., Basquin, J. & Griesbeck, O. Targeted In Situ Protein Diversification and Intra-organelle Validation in Mammalian Cells. *Cell Chem Biol* **27**, 610-621 e615 (2020).
